# Effect of Autoclave Sterilization on the Number of Uses and Resistance to Cyclic Fatigue of WaveOne Gold and Four Replica-Like Endodontic Instruments

**DOI:** 10.1155/2024/6628146

**Published:** 2024-05-29

**Authors:** Gustavo Ragozzini, Amjad Abu Hasna, Fernando Antonio Siano dos Reis, Felipe Bernardo de Moura, Tiago Moreira Bastos Campos, Carlos Eduardo Silveira Bueno, Cláudio Antonio Talge Carvalho, Alexandre Sigrist de Martin

**Affiliations:** ^1^ Faculdade São Leopoldo Mandic Instituto de Pesquisas São Leopoldo Mandic, Endodontia, Campinas, São Paulo, Brazil; ^2^ Department of Restorative Dentistry Endodontics Division Institute of Science and Technology São Paulo State University (ICT-UNESP), Eng. Francisco José Longo Avenue 777, São José dos Campos, São Paulo CEP 12245-000, Brazil; ^3^ School of Dentistry Universidad Espíritu Santo, Samborondón, Ecuador; ^4^ Physics Department Aeronautics Technological Institute (ITA), São José dos Campos, São Paulo, Brazil

## Abstract

This study aimed to evaluate the effect of autoclave sterilization on the integrity and instruments' fracture number after multiple uses and cyclic fatigue of the original WaveOne Gold (Dentsply Sirona Endodontics) compared to four replica-like instruments (TF4-Gold, Roll-Wave-Gold, W-File, and Micro-Gold). The instruments were analyzed by scanning electron microscope (SEM) before being used in root canal instrumentation (baseline). One hundred and fifty human molars, freshly extracted for orthodontic reasons or periodontal disease and with severe curvature (between 30° and 60°), were used. Fifty teeth were instrumented with 10 instruments from each group and were evaluated for integrity. After sterilization in an autoclave, the instruments were analyzed by SEM. This procedure was repeated twice more, totaling three rounds of instrumentation, sterilization, and SEM analysis. Ten unused instruments from each group were evaluated for resistance to cyclic fatigue in a static test using a motor and a device simulating a canal with a 60° curvature angle. The instruments were driven by the motor until separation, visually verified, and the time measured in seconds. Data were analyzed by *Chi*-square, one-way ANOVA, and Tukey analysis, considering a significance level of 5%. It was found that there was no statistically significant difference between the groups tested in the effect of sterilization on the number of uses. The SEM analysis showed distortions in the instruments after the 3rd use. There was a statistically significant difference in the cyclic fatigue test between the results of WaveOne Gold, TF4 Gold, and Roll Wave Gold compared to W File and Micro Gold (*P*  < 0.0001) and a statistically significant difference between the W File and Micro Gold groups (*P*  < 0.0001). In conclusion, this study affirmed that WaveOne Gold, TF4-Gold, and Roll-Wave-Gold instruments exhibit comparable cyclic fatigue resistance. Besides, all examined instruments can be reliably employed for up to two cases.

## 1. Introduction

Cleaning and shaping of the root canal system are essential to achieve the biological and mechanical goals of endodontic treatment by removing pulp tissue, bacteria, and their byproducts and providing adequate conformation for obturation [1, 2, 3]. Nickel–Titanium (NiTi) instruments have improved the instrumentation quality in endodontics compared to stainless steel instruments, as Ni–Ti has greater flexibility, greater resistance to torsion fracture, better-cutting efficiency, shorter preparation time, and better ability to centralize the instrumentation [4, 5, 6]. However, some adverse effects are still observed with Ni–Ti instruments, such as instrument fracture, step formation, and perforations [7, 8, 9, 10].

Some improvements were incorporated in the use of Ni–Ti instruments, such as the reciprocating kinematics that extend the useful life of the instrument, providing an increase in its resistance to fatigue [11] and reducing the occurrence of plastic deformation [12]. Besides, new surface treatments, including the thermal one [13], have allowed the development of instruments with a crystalline structure in intermediate stages, between the austenitic and martensitic phases, with substantial stability of the martensitic phase at body temperature, influencing its mechanical properties, since the martensitic phase has greater elasticity and can achieve greater deformation with relatively low stresses compared to the austenitic phase [14].

The WaveOne Gold (Dentsply Sirona Endodontics, Baillagues, Switzerland) is an original instrument, with reciprocating kinematics and is composed of considerable amounts of martensite, obtained by heat treatments of the NiTi alloy. Currently, a wide variety of instruments with different characteristics (design, cross-section, heat treatments, kinematics, etc.) [15, 16] have been launched on the market with the aim of improving the quality of endodontic treatments and reducing the possibility of accidents and/or complications [17, 18, 19]. The manufacture of these instruments must follow a sequence of research, development, and production tests before commercialization with controlled quality standards. However, several companies started to produce and distribute instruments with similar characteristics to systems of well-known brands without the publication of accurate reports on quality control, production, or international certification [20].

These instruments, so-called replica-like, have similar characteristics to the originals, such as nomenclature, color identification, number, and sequence [21], and have been sold worldwide over the internet or by local distributors with relatively lower prices than the originals, making them attractive for professionals [22]. Nonetheless, the clinical safety and efficiency of most of these systems have yet to be confirmed from a scientific point of view.

Examples of replica instruments include TF4 Gold (Shenzhen Perfect Medical Instruments, Shenzhen, China), Roll Wave Gold (Shenzhen Denco Medical, Shenzhen, China), W File (TDK, Shenzhen, China), and Micro Gold ((Microdont, Shenzhen, China).). The clinical safety and efficiency of most of these replica instruments have not yet been confirmed from a scientific point of view as they do not have published accurate reports on quality control, production, or international certification.

To date, within the researched literature, studies on the mechanical behavior of replica-type systems are rare. The present study aimed to evaluate the effect of autoclave sterilization on (I) the integrity after varied uses of four replica-like instruments in comparison with the WaveOne Gold (Dentsply Sirona Endodontics, Baillagues, Switzerland) and (II) the number of fractures, besides (III) the performance of these instruments in a static test of cyclic fatigue.

The null hypothesis under investigation posited that, following three cycles of autoclave sterilization and root canal instrumentation, the replica instruments would demonstrate equivalence to the original instrument in terms of fracture numbers. Additionally, it proposed that the instruments would exhibit equivalence with respect to cyclic fatigue in static testing.

## 2. Materials and Methods

### 2.1. Sample Size Calculation

Considering the mean and standard deviation of the experimental groups of [15], using the site https://www.sealedenvelope.com, in which the significance level *α* = 5%, the power (1−*β*) = 80%, standard deviation = 143.7, and equivalence limit = 210, it was found that the sample size required per group = 10.

### 2.2. Specimens' Selection

This study involved the collection of 150 newly extracted molars, which were obtained for orthodontic reasons or due to periodontal disease. Ethical approval for the study was granted by the Research Ethics Committee of the Institute of Science and Technology of São Paulo State University (approval number: 5.940.123); in addition, informed consents were obtained from all subjects and/or their legal guardian. In this study, all methods were performed in accordance with the relevant guidelines and regulations.

Following extraction, the teeth underwent disinfection using 1% sodium hypochlorite (NaOCl) for 24 hr. Subsequently, they were rinsed with saline solution and preserved by freezing until their utilization.

Inclusion criteria of the study:

1. Noncarious molars;
2. Hydrated condition;
3. Fully formed apex;
4. Absence of calcification;
5. Untreated upper or lower molars with significant curvature (30°–60°) according to Schneider's method [23].

Conversely, molars with caries, molars that were not properly hydrated, molars with incompletely formed apices, molars showing signs of calcification, previously endo-treated molars, or any molars with a curvature outside the range of 30°–60° were excluded from the study.

### 2.3. Experimental Groups

Five different endodontic systems (*n* = 10) were evaluated in this study:

1. WaveOne Gold (#25.07) of Dentsply (Dentsply Sirona Endodontics, Baillagues, Switzerland);
2. TF4 Gold (#25.07) of Dental Perfect (Shenzhen Perfect Medical Instruments, Shenzhen, China);
3. Roll Wave Gold (#25.07) of Denco (Shenzhen Denco Medical, Shenzhen, China);
4. W File (#25.07) of TDK (TDK, Shenzhen, China); and
5. Micro Gold (#25.07) of Microdont (Microdont, Shenzhen, China).

### 2.4. Scanning Electron Microscope (SEM)

As explained in detail in a previous study [24], two instruments of each brand were examined under an SEM (Inspect S50, PT Multi Teknindo Infotronika, Jakarta, Indonesia) to assess any fractures or cracks (baseline assessment). The instruments were cleaned with absolute alcohol for 3 min, fixed on a metal stub, and examined at high magnifications (100x, 200x, 500x, 1,000x, 2,000x, and 5,000x). After this initial evaluation, the topographic features of the surfaces of these instruments were evaluated three more times after each instrumentation and autoclave sterilization cycle.

### 2.5. Autoclave Sterilization Effect on the Number of Uses

The study involved a collection of 150 molars (450 canals), and these specimens underwent conventional endodontic access cavities with a 1,014-long shank drill (KG Sorensen) performed by a single operator who was an endodontist. Then the pulp chamber roof was removed by an Endo Z bur (Angelus, Lodrina, PR, Brazil), followed by smoothing and finishing the access cavity. The canals were then explored using #10 stainless steel K-files (Dentsply Maillefer, Oklahoma, USA), with the working length determined to be 1 mm short of the apical foramen.

The teeth were evaluated by periapical radiographs, separated according to the degree of root canal curvature (5°–19° as mild curvature; 20°–39° moderate curvature, and 40°–60° severe curvature) and randomly distributed into five groups, according to the instruments used: WaveOne Gold #25.07 (Dentsply Sirona); TF4 Gold #25.07 (Shenzhen Perfect); Roll Wave Gold #25.07 (Shenzhen); W File #25.07 (TDK, Shenzhen, China) and Micro Gold #25.07 (Microdont).

**Figure 1:**
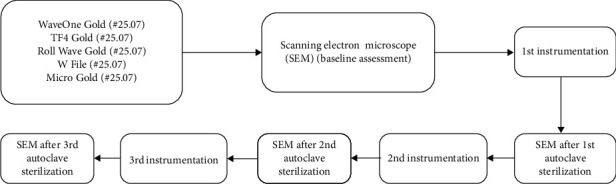
Flowchart of the instrumentation, SEM, and autoclave sterilization cycles.

Using 10 instruments of each brand (*n* = 10), biomechanical preparation was carried out on 50 molars (*n* = 10) following the manufacturer's guidelines, using a VDW Silver endodontic motor (VDW, Munich, Germany) adjusted to factory reciprocating kinematics (WAVEONE ALL). Biomechanical preparation was carried out in three phases, dividing the root canal into three thirds (cervical, middle, and apical) with short entry and exit movements. Sodium hypochlorite (2.5% NaOCl was used as the endodontic irrigant, with 5 mL used for each third, summing up to 15 mL per canal). This standardized protocol was applied to all root canals. Following this, the instruments underwent thorough cleaning using an ultrasonic cleaner and were then sterilized in an autoclave (Vitale Plus 21 Cristofoli Biosafety Equipment, Campo Mourão, PR, Brazil). Subsequently, the instruments underwent another round of SEM analysis. This entire process of instrumentation, autoclave sterilization, and SEM analysis was repeated three times, with each iteration involving 50 teeth, culminating in a total of 150 instrumented molars.  Figure 1 shows the flowchart of the experimental procedures carried out in this study. All of this sequence was followed as reported in the literature by another study [24].

### 2.6. Autoclave Sterilization Procedure

As reported in the literature, the protocol of the autoclave was followed [24]. First, the instruments were cleaned using an ultrasonic cleaner and detergent for 15 min. Then, the instruments were rinsed abundantly with running water and then dried with absorbing paper. Subsequently, they were meticulously placed into autoclave bags or pouches specifically designed for sterilization. The pouches were then sealed according to the manufacturer's instructions. Once properly packaged, the pouches were arranged within the autoclave chamber, with careful attention to prevent overcrowding. This arrangement ensured sufficient spacing between pouches, facilitating uniform steam penetration for effective sterilization. The autoclave settings were configured in accordance with the manufacturer's guidelines, utilizing steam under a pressure of 30 psi and a temperature range of 121−134°C. The recommended sterilization duration was 15 min.

Upon completion of the autoclave cycle, which included heating, sterilization, and cooling phases, the sterilized instruments were carefully removed from the chamber. To preserve their sterility, they were stored in a clean, dry, and secure environment. This meticulous process ensures that the instruments are prepared for safe and sanitary use.

### 2.7. Cyclic Fatigue

**Figure 2:**
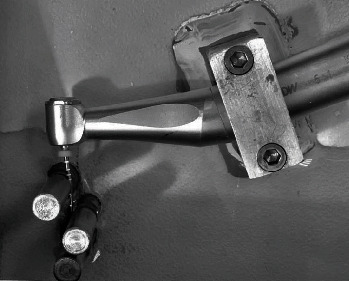
The instrument, while activated in a 60°, simulated root canal curvature at a water temperature of 37 ± 0.5°C.

Ten different instruments of each brand were tested and coupled to the VDW Silver endodontic motor adjusted to factory reciprocating kinematics (WAVEONE ALL). A plastic base with three adjustable stainless-steel pins (6 mm in diameter, 4 cm in length, 0.5 mm in width V-notch) was employed to simulate root canal curvature of 60° in which the instruments were tested according to previous studies [15] (Figure 2). This setup was submerged in 200 mL of deionized water inside a heating chamber (Kasvi, Taiwan). The water temperature was set at 37 ± 0.5°C; during all tests, the temperature was measured with an infrared thermometer. This methodology was validated in different reports and studies in the literature [15, 24].

All instruments were tested at a bending radius of 3 mm with a bending angle of 60°. The center of curvature was 4.5 mm from the tip, and the working length was 19 mm for each instrument tested. The radius and angle of curvature were determined according to a previous study [25]. The instruments were systematically driven by a motor until fracture occurred, with precise time measurements recorded in seconds, providing data for analysis and comparison.

### 2.8. Statistical Analysis

The analysis of the number of uses data was conducted using *Chi*-squared analysis to determine the statistical significance of the differences observed. For the cyclic fatigue data, a one-way ANOVA was performed to compare the means of different groups, followed by Tukey's post hoc test to identify specific group differences. These comprehensive analyses were executed utilizing GraphPad Prism 5.0 software. A significance level of 5% (*P*  < 0.05) was predetermined for all tests to ensure the reliability of the results. This approach allowed for a robust statistical evaluation of the data, providing clear insights into the performance and durability of the instruments under study.

## 3. Results

### 3.1. Autoclave Sterilization Effects on a Number of Uses

Table 1 displays the count of fractured instruments following the 1st, 2nd, and 3rd usage. The data in Table 1 reveals that there were no statistically significant differences observed among the groups subjected to testing.

### 3.2. SEM

**Figure 3:**
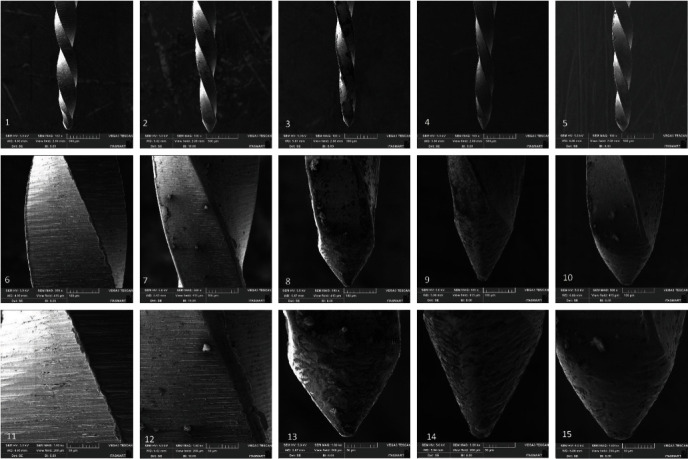
Scanning electron microscopy images of nonused instruments: WaveOne Gold at 100x (1), 500x (6), 1,000x (11), TF4 Gold at 100x (2), 500x (7), 1,000x (12), Roll Wave Gold at 100x (3), 500x (8), 1,000x (13), W file at 100x (4), 500x (9), 1,000× (14), and Micro Gold instrument, at 100x (5), 500x (10), 1,000x (15).

Within the WaveOne Gold group, the initial finish of the unused instrument was deemed satisfactory. Conversely, in the TF4 Gold, Roll Wave Gold, W File, and Micro Gold groups, the finishing was comparatively less refined when compared to the WaveOne Gold group, in which a few defects, such as porosities in the cutting edges and flutes, could be observed. SEM analysis revealed that all instruments subjected to testing displayed uncutting tips. However, the tips were observed to be more rounded in the case of the WaveOne Gold group and resembled a triangular shape in all the other tested groups (Figure 3).

**Figure 4:**
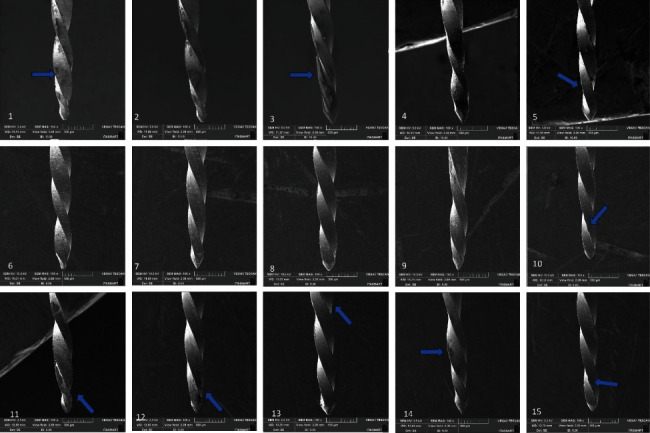
Scanning electron microscopy images of the tested instruments at 100×: WaveOne Gold 1st use (1), 2nd use (6), 3rd use (11), TF4 Gold 1st use (2), 2nd use (7), 3rd use (12), Roll Wave Gold 1st use (3), 2nd use (8), 3rd use (13), W file 1st use (4), 2nd use (9), 3rd use (14), and Micro Gold 1st use (5), 2nd use (10), 3rd use (15). Blue arrows indicate distortions.

Moreover, distortions were identified in specific instances: in the TF4 Gold group, distortions emerged following the third usage; in the Roll Wave Gold group, similar distortions were noted after the first, second, and third usages; for the W File group, distortions were observed after the third usage; and finally, in the Micro Gold group, distortions were apparent after the first, second, and third usages (Figure 4).

### 3.3. Cyclic Fatigue

**Figure 5:**
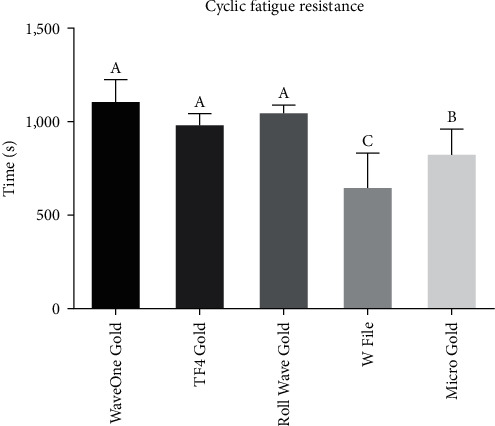
The cyclic fatigue resistance (time to fracture in seconds) of the tested groups. Different uppercase letters indicate statistically significant differences.

The analysis revealed that no statistically significant difference existed among the evaluated groups: WaveOne Gold, TF4 Gold, and Wave Roll Gold. However, these groups displayed a significant statistical difference when compared to the W File and Micro Gold groups (*P*  < 0.0001). Notably, a significant statistical difference was also observed between the W File and Micro Gold groups (*P*  < 0.0001) (Figure 5).

## 4. Discussion

Since the introduction of Gold heat treatment in endodontics by Dentsply Sirona Endodontics, many other companies have created “replica-like” instruments that have undergone similar heat treatments [26]. Some examples of these systems are the TF4 Gold (Shenzhen Perfect Medical Instruments, Shenzhen, China), Roll Wave Gold (Shenzhen Denco Medical, Shenzhen, China), W file (TDK, Shenzhen, China), and Micro Gold (Microdont, Shenzhen, China). However, currently, there is little literature describing their clinical and mechanical performance, efficacy, and safety [19]. Thus, it becomes urgent to know the real characteristics of these instruments to provide an answer to these questions. Therefore, in this study, aspects such as manufacturing quality were evaluated through analysis by SEM, the effect of autoclave sterilization on the number of uses of the instruments, and the cyclic fatigue using the original WaveOne Gold system as control. The first null hypothesis of this study was accepted; there was equivalent behavior of the replica instruments with the original in terms of fracture numbers. However, the second null hypothesis was not accepted, and the instruments demonstrated a statistically significant difference in fracture times in the cyclic fatigue test.

The surface characteristics of Ni–Ti instruments must be considered when evaluating instrument quality, as several conditions can affect corrosion resistance [27]. Surface analysis can be performed using SEM, the most widely used method for this. However, this methodology has the disadvantage of allowing only qualitative assessments, as the 2D images created cannot be processed for quantitative surface data [28]. The initial SEM analysis of the instruments revealed dissimilarities in the surfaces of the instruments, with greater porosity being observed in the TF4 Gold, Roll Wave Gold, W file, and Micro Gold systems, suggesting possible differences in metallurgical processing methods. Regarding the design of the instruments, it was possible to verify that they were similar, except for the geometry of the tips with an inactive guide. In the WaveOne Gold group, less irregularities were observed in the instrument; however, a distortion was found after the first use. Distortions were also found in the TF4 Gold, Roll Wave Gold, and Micro Gold groups after the first, second, and third uses and in the W File group after the third use.

The term cyclic fatigue describes the breakdown of Ni–Ti instruments after continuous rotation in a curved root canal, occurring because of subjecting the instruments to alternating cycles of stress compression when flexed in the region of maximum curvature of the root canal [29]. During this test, the longest time-to-fracture among all tested instruments was observed with the WaveOne Gold (1,094 s) and the shortest with the W file (636 s), followed by the Micro Gold (812 s), TF4 Gold (971.3 s) and Roll Wave Gold (1,033 s). The greater surface roughness could explain the lower resistance to cyclic fatigue of W file and Micro Gold compared to other instruments. It is important to point out that there is no detailed information on the instruments' manufacturing process in the literature or on the brands' websites.

The cyclic fatigue test does not have particular standards; however, over the years, some modifications have been proposed in an attempt to mimic the clinical conditions [30]. Although carrying out the cyclic fatigue test under the same experimental conditions allows controlling the interference of several variables, thus increasing its internal validity, several innovative discoveries would never have been carried out without the variation of a certain configuration [31]. In the present study, it was not possible to compare the results of the cyclic fatigue test with other studies, as this is the first article in the literature studying the mechanical performance of the tested systems, according to the researched literature.

Regarding the effects of sterilization on the number of uses of the instruments, no statistical difference was observed between the groups evaluated in this study. Only the Micro Gold group presented fractures on the second use, and the other groups presented fractures after the third use. This demonstrates that the systems would be safe to use in up to two clinical cases, which is in line with previous studies that indicate that the use of reciprocating instruments in multiple cases can be stable and efficient [32]. It is important to emphasize that the manufacturers recommend that the instruments be used only once, reducing the risk of accidents. Some studies have shown that repeated sterilization increases the surface roughness of Ni–Ti instruments [33], as well as reduces their cutting efficiency [30]. These surface alterations can lead to instrument fractures during root canal preparation, compromising the result of treatment [34].

Conversely, this study noted a decrease in surface irregularities after the initial use and the first sterilization cycle for all groups, as observed in the initial SEM analysis. This is in line with previous studies in the literature [35, 36]. Van-Pham's study [36] highlighted reduced porosity on the surfaces of Wave One Gold and Reciproc Blue after root canal preparation, potentially attributed to instrument wear during use. The fine polishing of contact areas in instruments exposed to lighter loads is believed to contribute to this phenomenon [36].

In this study, the cyclic fatigue was evaluated at simulated body temperature, while the instruments were driven by the motor, until the fracture, as the surrounding temperature influences the cyclic fatigue resistance and a deflecting load of the superelastic Ni–Ti instruments according to the literature [37].

From the literature consulted, a few articles investigated the TF4 Gold, Roll Wave Gold, W file, and Micro Gold systems used in this work; thus, more studies should be carried out to help elucidate the safety of the clinical use of these instruments, as well as checking its dimensions, design, and heat treatment procedures. Therefore, within the constraints of this study, it was found that the TF4 Gold and Roll Wave Gold replica-like instruments were similar to WaveOne Gold in terms of resistance to cyclic fatigue. Regarding the effect of autoclave sterilization on the number of uses, the replica-like instruments were similar to the original system. WaveOne Gold replica instruments appear to be expected for use in up to two cases. However, further studies are needed to confirm these findings.

This in vitro study, while valuable, carries inherent limitations as it does not fully replicate clinical conditions. Consequently, the findings should be substantiated by subsequent clinical investigations. Recognizing that the controlled laboratory environment may not entirely mirror real-world scenarios, caution is warranted in directly extrapolating the results to clinical practice. Future research involving clinical studies is crucial for providing a more comprehensive understanding and validation of the outcomes observed in this controlled setting.

## 5. Conclusions

1. Autoclave sterilization after successive uses of the tested instruments did not result in a decrease in their resistance.
2. All instruments tested appear to be reliable for use in up to two cases.
3. WaveOne Gold, TF4 Gold, and Roll Wave Gold showed similar behavior in relation to resistance to cyclic fatigue.

## Figures and Tables

**Table 1:** The number of fractures per use.

	Fractures (1st use)	Fractures (2nd use)	Fractures (3rd use)	Total fractures
WaveOne Gold	0	0	2	2
TF4 Gold	0	0	2	2
Roll Wave Gold	0	0	2	2
W File	0	0	2	2
Micro Gold	0	1	2	3

## Data Availability

The data used to support the findings of this study are available upon request with the corresponding author mailto: d.d.s.amjad@gmail.com.

## References

[1] Schilder H. (1974). Cleaning and shaping the root canal. *Dental Clinics of North America*.

[2] Carvalho A. S., de Oliveira L. D., Cardoso F. G. R., de Oliveira F. E., Valera M. C., Carvalho C. A. T. Limewater and polymyxin B associated with NaOCl for endotoxin detoxification in root canal with necrotic pulp. *Brazilian Dental Journal*.

[3] de Oliveira L. D., de Oliveira F. E., Hatje B. A., Valera M. C., Carvalho C. A. T., Hasna A. A. Detoxification of LTA by intracanal medication: analysis by macrophages proinflammatory cytokines production. *Brazilian Dental Journal*.

[4] Walia H. M., Brantley W. A., Gerstein H. (1988). An initial investigation of the bending and torsional properties of Nitinol root canal files. *Journal of Endodontics*.

[5] Kazemi R. B., Stenman E., Spngberg L. S. W. (1996). Machining efficiency and wear resistance of nickel–titanium endodontic files. *Oral Surgery, Oral Medicine, Oral Pathology, Oral Radiology, and Endodontology*.

[6] Bishop K., Dummer P. M. (1997). A comparison of stainless steel flexofiles and nickel–titanium NiTiFlex files during the shaping of simulated canals. *International Endodontic Journal*.

[7] Yared G. (2008). Canal preparation using only one Ni–Ti rotary instrument: preliminary observations. *International Endodontic Journal*.

[8] Gutmann J. L., Gao Y. (2012). Alteration in the inherent metallic and surface properties of nickel–titanium root canal instruments to enhance performance, durability and safety: a focused review. *International Endodontic Journal*.

[9] Fišerová E., Chvosteková M., Bělašková S., Bumbálek M., Joska Z. (2015). Survival analysis of factors influencing cyclic fatigue of nickel–titanium endodontic instruments. *Advances in Materials Science and Engineering*.

[10] Al-Nahlawi T., Ala Rachi M., Abu Hasna A. (2021). Endodontic perforation closure by five mineral oxides silicate-based cement with/without collagen sponge matrix. *International Journal of Dentistry*.

[11] De-Deus G., Moreira E. J. L., Lopes H. P., Elias C. N. Extended cyclic fatigue life of F2 ProTaper instruments used in reciprocating movement. *International Endodontic Journal*.

[12] De-Deus G., Cardoso M. L., Simões-Carvalho M. (2021). Glide path with reciprocating driven pathfinding instrument: performance and fracture rate. *Journal of Endodontics*.

[13] Vieira T. M., Cardoso R. M., A/lves N. C. C. (2021). Cyclic fatigue resistance of blue heat-treated instruments at different temperatures. *International Journal of Biomaterials*.

[14] Gavini G., Santos M. D., Caldeira C. L. (2018). Nickel–titanium instruments in endodontics: a concise review of the state of the art. *Brazilian Oral Research*.

[15] Camargo C. H. R., Bittencourt T. S., Hasna A. A., Palo R. M., Carvalho C. A. T., Valera M. C. (2020). Cyclic fatigue, torsional failure, and flexural resistance of rotary and reciprocating instruments. *Journal of Conservative Dentistry and Endodontics*.

[16] Riyahi A. M., Bashiri A., Alshahrani K., Alshahrani S., Alamri H. M., Al-Sudani D. (2020). Cyclic fatigue comparison of trunatomy, twisted file, and protaper next rotary systems. *International Journal of Dentistry*.

[17] Zupanc J., Vahdat-Pajouh N., Schäfer E. (2018). New thermomechanically treated NiTi alloys—a review. *International Endodontic Journal*.

[18] de Almeida B. C., Elias C. N. (2020). Influence of heat treatment on color and flexibility of nickel-titanium endodontic instruments. *RGO—Revista Gaúcha de Odontologia*.

[19] Chan W.-S., Gulati K., Peters O. A. (2023). Advancing Nitinol: from heat treatment to surface functionalization for nickel–titanium (NiTi) instruments in endodontics. *Bioactive Materials*.

[20] Martins J. N. R., Silva E. J. N. L., Marques D. (2020). Mechanical performance and metallurgical features of protaper universal and 6 replicalike systems. *Journal of Endodontics*.

[21] Martins J. N. R., Silva E. J. N. L., Marques D. (2020). Influence of kinematics on the cyclic fatigue resistance of replicalike and original brand rotary instruments. *Journal of Endodontics*.

[22] Logsdon J., Dunlap C., Arias A., Scott R., Peters O. A. (2020). Current trends in use and reuse of nickel–titanium engine-driven instruments: a survey of endodontists in the United States. *Journal of Endodontics*.

[23] Schneider S. W. (1971). A comparison of canal preparations in straight and curved root canals. *Oral Surgery, Oral Medicine, Oral Pathology*.

[24] Dos Reis F. A. S., Abu Hasna A., Ragozzini G. (2023). Assessing the cyclic fatigue resistance and sterilization effects on replica-like endodontic instruments compared to reciproc blue. *Scientific Reports*.

[25] de Vasconcelos R. A., Murphy S., Carvalho C. A. T., Govindjee R. G., Govindjee S., Peters O. A. (2016). Evidence for reduced fatigue resistance of contemporary rotary instruments exposed to body temperature. *Journal of Endodontics*.

[26] Martins J. N. R., Silva E. J. N. L., Marques D. (2022). Design, metallurgical features, and mechanical behaviour of NiTi endodontic instruments from five different heat-treated rotary systems. *Materials*.

[27] Schäfer E. (2002). Effect of sterilization on the cutting efficiency of PVD-coated nickel–titanium endodontic instruments. *International Endodontic Journal*.

[28] Sağlam B. C., Görgül G. C. (2015). Evaluation of surface alterations in different retreatment nickel–titanium files: AFM and SEM study. *Microscopy Research and Technique*.

[29] Sattapan B., Nervo G. J., Palamara J. E., Messer H. H. (2000). Defects in rotary nickel–titanium files after clinical use. *Journal of Endodontics*.

[30] Hülsmann M., Donnermeyer D., Schäfer E. (2019). A critical appraisal of studies on cyclic fatigue resistance of engine-driven endodontic instruments. *International Endodontic Journal*.

[31] Peters O. A., Arias A., Choi A. (2020). Mechanical properties of a novel nickel–titanium root canal instrument: stationary and dynamic tests. *Journal of Endodontics*.

[32] Pirani C., Paolucci A., Ruggeri O. (2014). Wear and metallographic analysis of WaveOne and reciproc NiTi instruments before and after three uses in root canals. *Scanning*.

[33] Yılmaz K., Uslu G., Özyürek T. (2018). Effect of multiple autoclave cycles on the surface roughness of HyFlex CM and HyFlex EDM files: an atomic force microscopy study. *Clinical Oral Investigations*.

[34] Valois C. R. A., Silva L. P., Azevedo R. B. (2008). Multiple autoclave cycles affect the surface of rotary nickel-titanium files: an atomic force microscopy study. *Journal of Endodontics*.

[35] Ba-Hattab R., Almohareb R. A., Alkhalaf R., Binnjefan S., Sulayem M., Barakat R. M. (2022). The impact of multiple autoclave cycles on the surface roughness of thermally treated nickel–titanium endodontic files. *Advances in Materials Science and Engineering*.

[36] Van Pham K., Vo C. Q. (2020). A new method for assessment of nickel-titanium endodontic instrument surface roughness using field emission scanning electronic microscope. *BMC Oral Health*.

[37] Jamleh A., Yahata Y., Ebihara A., Atmeh A. R., Bakhsh T., Suda H. (2016). Performance of NiTi endodontic instrument under different temperatures. *Odontology*.

